# Adjuvant sequential methotrexate → 5-fluorouracil *vs* 5-fluorouracil plus leucovorin in radically resected stage III and high-risk stage II colon cancer

**DOI:** 10.1038/sj.bjc.6602276

**Published:** 2004-12-21

**Authors:** A Sobrero, G Frassineti, A Falcone, L Dogliotti, R Rosso, F D Costanzo, P Bruzzi

**Affiliations:** 1 Oncologia Medica Ospedale S Martino, Largo Benzi 10, Genova 16132, Italy; 2 Istituto Oncologico Romagnolo, Forli, Italy; 3 Oncologia medica, Ospedale di Livorno, Italy; 4 Cattedra di Oncologia Medica, Universita' di Torino, Italy; 5 Istituto Nazionale per la Ricerca sul Cancro, Genova, Italy; 6 Oncologia Medica Ospedale di Careggi, Firenze, Italy

**Keywords:** methotrexate, 5-fluorouracil, leucovorin, adjuvant, colorectal cancer

## Abstract

The aim of the study was to determine whether modulation of 5-fluorouracil (FU) by methotrexate (MTX) improves survival compared to FU+6-*s*-leucovorin (LV) following potentially curative resection of stage II and III colon cancer. Within 8 weeks from surgery, 1945 patients with stage III (44%) or high-risk stage II (55%) colon cancer were randomly assigned to receive either 6 monthly cycles of FU 370 mg m^−2^ i.v. bolus preceded by LV 100 mg m^−2^ i.v. bolus on days 1–5, or 6 monthly cycles of sequential MTX 200 mg m^−2^ i.v. days 1 and 15 and FU 600 mg m^−2^ i.v. on days 2 and 16 followed by LV rescue (15 mg given p.o. q 6 h × 6 doses). Levamisole 50 mg p.o. t.i.d. on days 1–3, every 14 days for 6 months, was planned to be given in both arms. After a median follow-up of 4.2 years, 568 patients have relapsed and 403 have died. Survival was similar with MTX → FU and FU+LV (77 *vs* 77% at 5 years; *P*=0.90), as were 5-year disease-free survivals (67 *vs* 63%; *P*=0.44). Efficacy results were similar for both stage III and II patients. There were two toxic deaths, two in the MTX → FU arm (0.2%) and zero in the control arm. We conclude that biochemical modulation of FU with LV or with MTX produces similar results in the adjuvant setting of colon cancer.

A series of improvements have been made in the adjuvant treatment of node-positive colon cancer. The first was the demonstrated efficacy of 12 months of 5-fluorouracil (FU) levamisole in 1990 (Moertel *et al*, 1990) and of FU 6-*s*-leucovorin (LV) in 1993 (Wolmark *et al*, 1993), and the second was the proven equivalence of only 6 months of FU LV to the standard 12 months of FU levamisole, 5 years later (Haller *et al*, 1998). The third was the demonstration that the benefit of adjuvant chemotherapy extends to elderly patients as well (Sargent *et al*, 2001). And the last advancement regards the additional substantial benefit that the doublet FU+oxaliplatin provides as compared to optimal FU chemotherapy (Andre *et al*, 2004). Other issues have been addressed and definitely answered by large-scale randomised trials in the last 5 years. Such is the case for the optimal dose of LV to potentiate FU: no need for high dose (Haller *et al*, 1998; Quasar, 2000); the best schedule of FU LV: equivalence between the weekly and the monthly schedule (Kerr, 2000), the lack of efficacy of levamisole (Haller *et al*, 1998; Quasar, 2000), the equivalence of the bolus and infusional FU regimens (Andre *et al*, 2003) and the inefficacy of short-term early postoperative intraportal FU infusions (Liver infusion meta-analysis group, 1997).

INTACC is an Italian intergroup that has recently reported another large-scale randomised trial of adjuvant chemotherapy for colon cancer comparing FU levamisole *vs* FU LV levamisole (Di Costanzo *et al*, 2003). When the accrual of that first study was completed in February 1995, levamisole was still considered part of the standard adjuvant treatment. We postulated that our experimental arm in the first study was unlikely to be inferior to the standard arm and used that arm, FU LV levamisole, given for 6 months as control of the second study. The experimental arm was decided to be the sequential combination of methotrexate (MTX) → FU (Marsh *et al*, 1991) along with levamisole. This choice was based on the results of the meta-analysis on MTX → FU *vs* FU in the advanced setting (Advanced colorectal cancer metaanalysis, 1994), suggesting that it was reasonable to test this combination in the adjuvant setting. The design of this trial seems definitely outdated in 2004, but at the time of planning, only an alternative modulation of FU other than LV could be feasible within our intergroup.

To our knowledge, this is the first reported study of adjuvant MTX → FU and we are unaware of any other such study ongoing. As we will discuss, some of the findings of this study may have important implications for the design of future adjuvant studies.

## PATIENTS AND METHODS

### Eligibility criteria

All patients were required to have had a histological proof of colon carcinoma above the peritoneal reflection, complete resection of the primary tumour without gross or microscopic evidence of residual disease, regional lymph node involvement and or transmural penetration of the muscular wall of the bowel with or without adherence or penetration into an adjacent organ (Dukes’ stages B2, B3 and C according to Modified Astler and Coller, 1987 classification). A minimum number of lymph nodes was not required by the protocol for defining a patient as node negative. (AJCC, 1987) ECOG performance status ⩽1, age ⩾18 years, normal hepatic and renal function and informed consent were also required.

The colon tumour was defined as any lesion of the large bowel which did not require the opening of the pelvic peritoneum to define the distal extent of the tumour and/or >12 cm from pectinate line but with the inferior margin of the tumour above the peritoneal reflection.

Patients were ineligible if they were pregnant or lactating, had had prior or concurrent radiation or chemotherapy for colon cancer, had had previous or concurrent second malignant disease (except superficial squamous or basal-cell skin carcinoma of the skin or *in situ* carcinoma of the cervix) or nonmalignant systemic disease precluding administration of chemotherapy. Those with WBC count less than 3500 *μ*l^−1^ and platelet count less than 100 000 *μ*L^−1^, were ineligible.

Patients were accrued from five central and northern Italian cooperative groups for a total of 71 centres: Gruppo Oncologico Italiano di Ricerca Clinica (GOIRC), Gruppo Cooperativo Istituto Nazionale per la Ricerca sul Cancro (Ist-Genova), Gruppo Oncologico del Nord-Ovest (GONO), Gruppo Oncologico Piemontese Tumori Apparato Digerente (GOPTAD) and Istituto Oncologico Romagnolo (IOR).

### Treatment

After stratification for centre, patients were randomly assigned to receive either 6 monthly cycles of FU 370 mg m^−2^ i.v. bolus preceded by LV 100 mg m^−2^ i.v. bolus on days 1–5 or 6 monthly cycles of sequential MTX 200 mg m^−2^ i.v. days 1 and 15 and FU 600 mg m^−2^ i.v. on days 2 and 16 followed by LV rescue (15 mg given p.o. q 6 h × 6 doses). No serum MTX level measurements were required on a routine basis. Levamisole 50 mg p.o. t.i.d. on days 1–3, every 14 days for 6 months. was given in both arms. The treatment was started within 60 days from surgery.

Toxicity was defined according to the World Health Organisation (WHO) criteria. Treatment was repeated at full doses if WBC was >3500 *μ*l and PLT >100 000 *μ*l; chemotherapy dose was delayed if WBC was <3500 *μ*l and/or PLT <100 000 *μ*l at recycle.

Chemotherapy was stopped for any grade (gr) 4 toxicity. FU was repeated at full dose in case of gr 1 diarrhoea or mucositis, whereas the dose was reduced by 25% for gr 2–3. Dose reduction of MTX, LV and levamisole were not allowed, since all these agents were used as modulators of FU. Investigators were required to notify the trial office by telephone of any serious unexpected adverse event.

### Follow-up

Forms were sent to the Data Centre of the study for each patient upon study entry and every 6 months for the first 3 years and yearly thereafter. Before each course of therapy, patients had to undergo a physical examination, haematologic and renal function studies and serum carcinoembryonic antigen. After completion of therapy, these tests were repeated every 6 months for the first 2 years and then every year for a total of 5 years.

During follow-up, colonoscopy or barium enema were recommended every 2 to 3 years. Chest X-ray and hepatic ultrasound or CT scan were performed every 6 months for the first 2 years, then every year until the 5th year. The treatment of relapse was not standardised and was left up to the investigators.

### Study end points and statistical methods

The main end point of the study was overall survival (OS); the secondary end point was DFS. A minimum sample size of 1500 patients (with annual accrual of 500 for 3 years) was set to ensure an 80% probability of detecting a 20% relative reduction in overall mortality after 5 years of follow-up, assuming a constant hazard rate equal to 8% per year (11) in the control arm, leading to an expected OS of 67% at 5 years.

Randomisation was centralised at the coordinating centres of Genova (for Genova, GONO and GOPTAD), GOIRC and IOR. Randomisation lists were generated with blocks of variable length in random sequence, unknown to clinicians. The clinical centre was the only stratification factor. All the analyses were carried out according to the intention-to-treat principle in that all randomised patients were included in the analyses. Overall survival was computed as the time from randomisation to death due to any cause. Disease-free survival (DFS) was computed as the time from randomisation to the first observation of disease relapse or occurrence of a second primary cancer, or death due to any cause. Kaplan–Meier estimate and log-rank test allowed comparisons among the curves. Overall survival and DFS analyses performed according to the treatment were stratified according to disease stage (TNM stage II and III). Cox's regression analysis was used to model OS and DFS as a function of a set of independent variables. All the variables were categorical: treatment assigned at randomisation (standard, experimental); ECOG performance status (0, 1); histological grading (1=well, 2=moderately well+missing values, 3=poorly differentiated; patients with missing values of grading were considered as G2 patients); TNM stage of disease (stage II, stage III with one to four positive nodes, stage III with ⩾5 positive nodes); gender; and site of disease (1=right colon: caecum, ascending colon and hepatic flexure; 2=left colon: transverse colon, splenic flexure and descending colon; 3=intraperitoneal rectum and sigmoid; 4=multiple locations). Age was used as a stratification factor. For Cox's regression analysis, a backward procedure was used with *P* in=0.05 and *P* out=0.10. All *P*-values were two-sided. SPSS 11.0 statistical package was used for all the analyses.

## RESULTS

**Figure 1:**
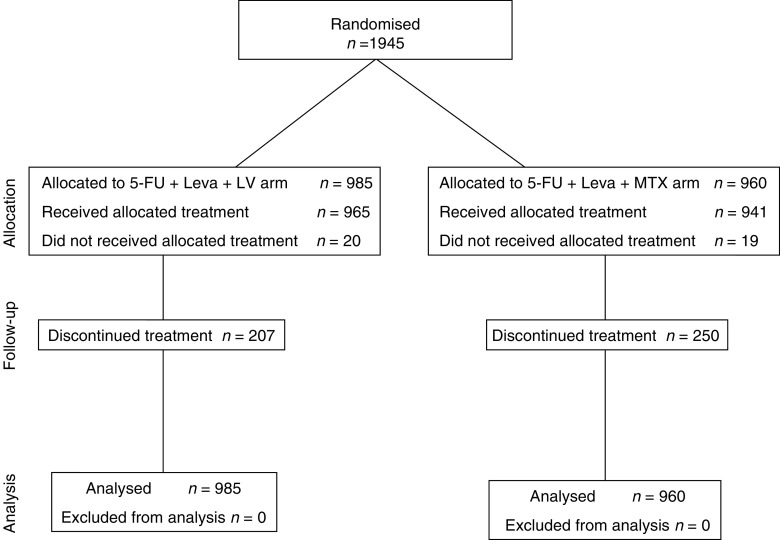
Consort diagram of the study.

Between March 1995 and February 1998, 1945 patients were accrued from 71 Italian centres (Consort diagram, Figure 1) and their characteristics are shown in Table 1. The median number of patients per centre was 19, but more than 50% of the patients were accrued from 14 centres. In terms of age group distribution, 17.0% were older than 70 years and 2.8% younger than 40 years. Among the protocol violations, eight patients had unknown PS, 19 patients were randomised after 60 days from surgery and 73 patients started chemotherapy longer than 8 weeks from surgery. In addition, 23 patients were ineligible for staging problems (five patients were stage I, one stage IV and 17 had no mention of the stage). About 55% patients were stage II and they were equally distributed in the two arms, which were well balanced in terms of the other major patients characteristics; among stage II and III, 91 had no information regarding lymph node status. In most of these cases, 74 of 91, the pathology report stated either ‘Dukes’ C’ or ‘Dukes’ B 2–3’, while 17 had missing stage information.

The median number of lymph nodes recovered according to the pathology report (*N*=1854 surgical operations) was 12 for stage II (range 0–74) and 12 for stage III patients (range 0–70).

The median time from surgery to randomisation and to the beginning of chemotherapy were 31 and 39 days, respectively.

### Compliance to treatment and toxicity

In all, 72% of the patients completed the planned six cycles of MTX → FU and 77% completed FU LV; approximately 12% discontinued the treatment after completing only three cycles (3 months) of therapy and 2% never started chemotherapy in either arm. No patients received more than the planned six cycles. Levamisole was never started in 22.2% of the experimental arm and in 21.7% of the control arm, and it was discontinued for toxicity, refusal or other reasons in another 20%, so that only 60% of patients received the immunomodulator according to the protocol. This was not an unexpected finding as this trial accrued patients when levamisole's efficacy was beginning to be challenged.

There were two toxic deaths in the MTX → FU and none in the control arm. One patient died of diarrhoea, dehydration and sepsis after the first cycle and the other of severe mucositis, diarrhoea and dehydration with shock. Overall, the two treatments had similar degrees of toxicity, although slightly different patterns (Table 2). Thrombocytopenia was more pronounced in the MTX → FU arm (2.2 *vs* 0.1%, gr 3 and 4). The same was true for mucositis (14.8 *vs* 11.7%), while the reverse occurred for diarrhoea (6.5 *vs* 12.6%, gr 3 and 4). Conjunctivitis of low grade (1 and 2) was twice more frequent in the experimental arm (21.2 *vs* 12.6%). Hand and foot syndrome of low grade occurred in 5% of FU LV- and in 2.8% of MTX → FU-treated patients (*P*=0.02). Neurotoxicity and hepatotoxicity was reported in fewer than 1% of cases in either arm.

### Efficacy

After a median follow-up time of 4.2 years, 198 (20.6%) deaths were observed in the experimental arm and 205 (20.5%) in the control arm; recurrences were 269 (28.0%) and 299 (30.3%) in the experimental and in the control arm, respectively. Univariate and multivariate hazard ratios are 0.94 (0.8–1.12) and 0.93 (0.78–1.1) for relapse, and 1.01 (0.83–1.23) and 1.03 (0.84–1.26) for overall mortality.

The sites of initial recurrence were not significantly different between arms; in patients treated with MTX → FU, they were the liver in 23.2%, other sites within the abdominal cavity in 20.2%, the lungs in 6.8% and multiple in 19.8%, while second primary tumours were reported in 5.7% of cases. Corresponding figures for the control arm were, respectively, 27.7, 17.9, 4.9, 21.4 and 5.3%. Aside from the two toxic deaths described, 37 deaths (10.4%) were unrelated to cancer, 22 (12.7%) in the experimental arm and 15 (8.2%) in the control arm.

**Figure 2:**
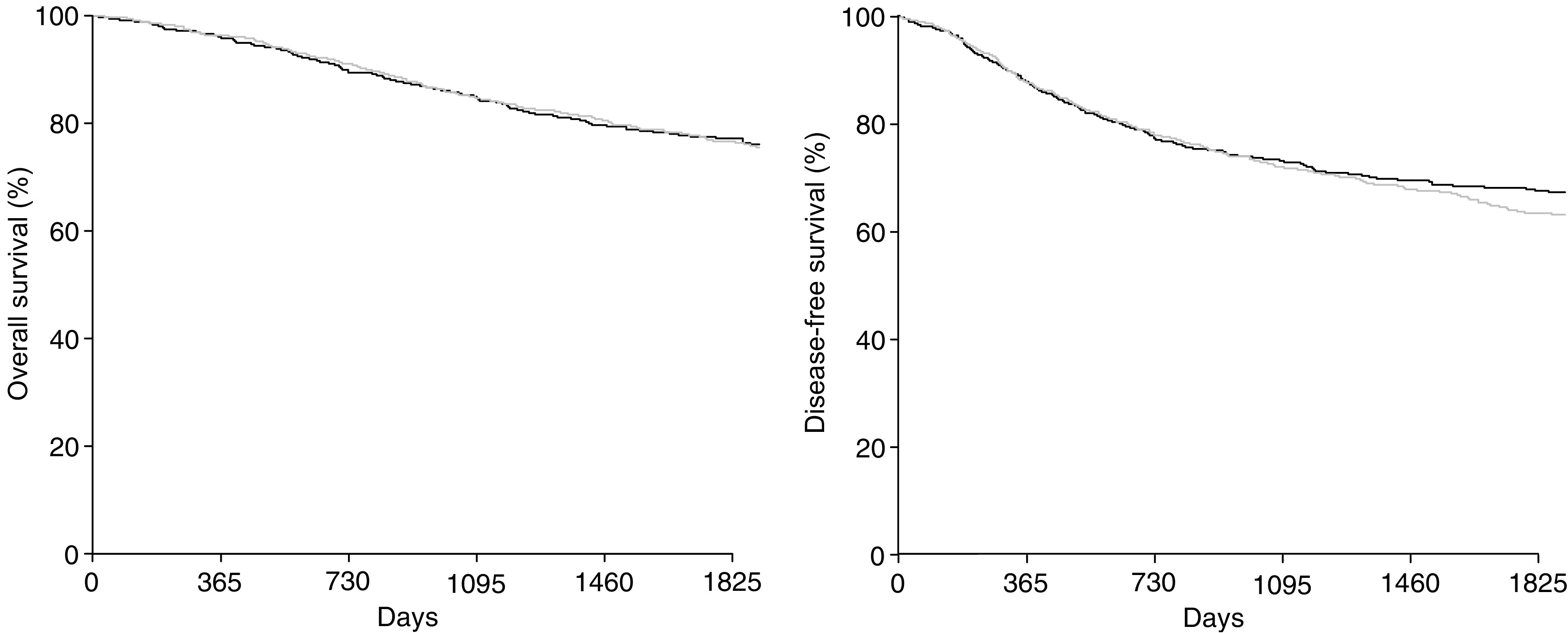
Overall survival and DFS of the entire patient population. Bold lines=MTX → FU levamisole; standard lines=FU LV levamisole.

**Figure 3:**
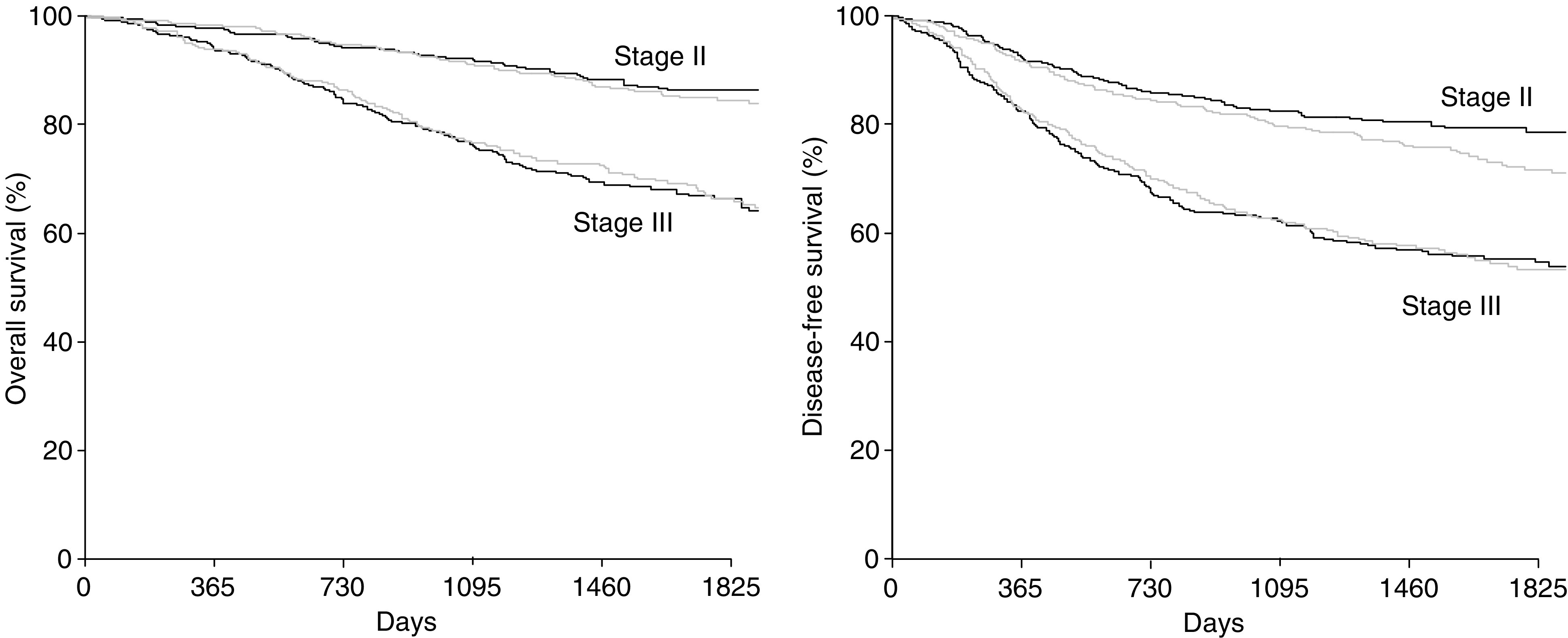
Overall survival and DFS as a function of stage. Bold lines=MTX → FU levamisole; standard lines=FU LV levamisole.

Figures 1 and 2 show that survival was similar with MTX → FU and FU+LV (77 *vs* 77% at 5 years; *P*=0.90), as were DFSs (67 *vs* 63% at 5 years; *P*=0.44). Efficacy results were similar for both stage III and II patients (Figure 3).

The hazard ratio for recurrence and death adjusted for stage were 0.99 (95% confidence limits=0.82–1.21) and 1.07 (95% confidence limits=0.96–1.19), respectively.

## DISCUSSION

At the time the study was designed, irinotecan and oxaliplatin were not available even for experimental use in the adjuvant setting and it was thought that comparing the sequential MTX → FU combination to standard FU LV (maintaining levamisole in both arms) was reasonable and appealing for a large-scale pragmatic trial for three reasons. First, while the meta-analysis on FU LV *vs* FU failed to show a significant survival benefit (Advanced colorectal cancer metaanalysis, 1992), the meta-analysis on MTX → FU did so, although the difference was small (Advanced colorectal cancer metaanalysis, 1994). Second, the meta-analysis on MTX → FU included several trials designed without the demonstrated optimal interval between the antifol and the fluoropyrimidine (4–24 h) leaving hope for more substantial efficacy of this sequence with appropriate scheduling, as we chose for our study. Third, while LV modulates FU by enhancing its anti-DNA effects via the inhibition of thymidylate synthase, MTX modulates FU primarily by increasing the incorporation of fluorouracil triphosphate into RNA, adding pharmacodynamic interest to the comparison (Sobrero *et al*, 1997). In 1995, all these good reasons appeared to justify this adjuvant trial.

Despite a certain percentage of missing data regarding patient and tumour characteristics, understandable in a large pragmatic trial, the overall good quality of colon cancer care in this trial is proven by two indirect indicators: the median number of lymph nodes removed at surgery, 12, and the very good 3-year DFS value, 74%. This value matches well with that of the control arm of the MOSAIC (Andre *et al*, 2004) study (73%), which in turn was identical to another European large study of adjuvant chemotherapy in this disease (Andre *et al*, 2003).

The large sample size of the study and the similarity of the OS and DFS curves in the two arms fail to demonstrate clinically relevant differences in efficacy between the regimens. Since the toxicity and duration of the two treatments are also similar, MTX → FU may be considered an alternative to FU+LV, but offers no advantage, even from the convenience standpoint.

The equivalence observed allows to speculate on the relevance of the total dose of FU in the adjuvant treatment of this disease. The classic FU levamisole used a total of 27 g m^−2^, the weekly NSABP regimen employed 18 g m^−2^ and in a later version 12 g m^−2^ and the monthly cycle between 11.1 g m^−2^ (as in the control arm of our trial) and 12.75 g m^−2^. Our MTX → FU regimen used only 7.2 g m^−2^. Despite its low activity as single agent against advanced disease, it is possible that MTX contributes as a cytotoxic to the efficacy of FU or that it is a better modulator than LV (as it was our hypothesis), thus accounting for the overall efficacy of so little FU. However, it is also likely that very little FU is needed for efficacy in sensitive patients. And these speculations may be the basis for treatment duration trials in this disease where the new, most efficacious FU+oxaliplatin combination cannot be tolerated by a large proportion of patients because of cumulative neurotoxicity.

Along the line of speculation, if only very little FU is sufficient, these data may provide additional rationale to identify the sensitive patient population. The recent work on TS, DCC, LOH 18q and MSI (Jen *et al*, 1994; Lenz *et al*, 1998; Halling *et al*, 1999; Gryfe *et al*, 2000; Watanabe *et al*, 2001) are good examples of such strategies. For FU-sensitive patients, very few courses of FU adjuvant chemotherapy may be sufficient, for the others, repeated cycles of FU administrations are likely to be entirely ineffective and FU might well be omitted from the adjuvant treatment programme in these patients.

## Figures and Tables

**Table 1:** Patient characteristics by treatment arm

	**5-FU+levamisole+MTX (960 pts)**		**5-FU+levamisole+LV (985 pts)**		**All patients (1945)**	
Median age (years) (range)	62 (30–83)		63 (25–80)		62 (25–83)	
*Gender* (%)						
Male	560	(58.3)	575	(58.4)	1135	(58.4)
Female	400	(41.7)	410	(41.6)	810	(41.6)
						
*ECOG performance status* (%)						
0	858	(89.8)	888	(90.4)	1746	(90.1)
1	97	(10.2)	94	(9.6)	191	(9.9)
Unknown	5		3		8	
						
*Stage* (%)						
Dukes B	517	(54.8)	543	(55.5)	1060	(55.2)
Dukes C	423	(45.2)	433	(44.3)	856	(44.8)
Other	14		9		23	
						
*Tumour location* (%)						
Right colon	256	(27.4)	311	(32.5)	567	(30.0)
Left colon	667	(71.5)	631	(65.8)	1298	(68.6)
Multiple locations	10	(1.1)	16	(1.7)	26	(1.4)
Unknown	27		27		54	
						
*Surgery* (%)						
Right hemicolectomy	289	(31.0)	336	(35.1)	625	(33.1)
Left hemicolectomy	298	(31.9)	292	(30.5)	590	(31.2)
Resection of sigmoid	169	(18.1)	174	(18.1)	343	(18.1)
Anterior resection	91	(9.8)	80	(8.4)	171	(9.0)
Other	86	(9.2)	76	(7.9)	162	(8.6)
Unknown	27		27		54	
						
*Histology* (%)						
Adenocarcinoma	918	(99.6)	951	(99.7)	1869	(99.6)
Undifferentiated	4	(0.4)	3	(0.3)	7	(0.4)
Unknown	38		31		69	
						
*Grade of anaplasia* (%)						
G1	84	(9.6)	70	(7.9)	154	(8.7)
G2	657	(75.2)	675	(75.9)	1332	(75.6)
G3	133	(15.2)	144	(16.2)	277	(15.7)
Unknown	86		96		182	

5-FU=5-fluorouracil; MTX=methotrexate; ECOG=Eastern Cooperative Oncology Group.

**Table 2:** Grade 3–4 toxicity (%)

	**5-FU+levamisole +MTX (960 pts)**	**5-FU+levamisole+LV (985 pts)**	***P*-value**
Leukopenia	35(4)	25(2.7)	0.16
Thrombocytopenia	19(2.2)	1(0.1)	<0.0001
Anaemia	8(0.9)	4(0.4)	0.39
Mucositis	131(14.8)	107(11.7)	0.048
Diarrhoea	57(6.5)	115(12.6)	<0.0001
Nausea and vomiting	31(3.5)	21(2.3)	0.12
Conjunctivitis	6(0.7)	2(0.2)	0.13
Hand–foot syndrome	2(0.2)	7(0.8)	0.12
