# Adjuvant sequential methotrexate → 5-fluorouracil *vs* 5-fluorouracil plus leucovorin in radically resected stage III and high-risk stage II colon cancer

**DOI:** 10.1038/sj.bjc.6602755

**Published:** 2005-09-13

**Authors:** A Sobrero, G Frassineti, A Falcone, L Dogliotti, R Rosso, F Di Costanzo, P Bruzzi

A Sobrero^1^, G Frassineti^2^, A Falcone^3^, L Dogliotti^4^, R Rosso^5^, F Di Costanzo^6^ and P Bruzzi^5^ on behalf of the INTACC^7^

**Correction to:** *British Journal of Cancer* (2004) **92**, 24–29. doi:10.1038/sj.bjc.6602276

Owing to an author error, the list of participating centres and collaborators was missed out of the above paper. The full list is given below. The author's apologise for this mistake.

INTACC Investigators are listed below:

Comandone A. Ospedale Gradenigo Torino; Bumma C, Ospedale S. Giovanni AS, Torino

Mestriner M, Ospedale S. Vito Torino, Faggiuolo R, IRCC Candiolo, Torino

Nanni O, Amadori D, Rosetti P, Ospedale Pierantoni Morgagni -Forli

Turci D, Marangolo M, Zumaglini F, Ospedale Santa Maria delle Croci-Ravenna

Boni C, Bisagni G, Banzi M, Ospedale di Reggio Emilia; Contu A, Olmeo N, Pazzola A, Ospedale di Sassari; Oliverio G, Venturini B, Ospedale degli Infermi-Rimini

Gambi A, Amaducci L, Ospedale degli Infermi-Faenza; Faedi M, Ospedale Bufalini-Cesena

Marzola M, Arcispedale S. Anna -Ferrara, Biasco G, Policlinico S. Orsola-Bologna

Antini M, Ospedale Sant Eugenio-Roma; Artioli F, Cagossi K, Gandini G, Ospedale Ramazzini-Carpi; Zuccarino L, Ospedale di Arzignano; Recchia F, Saggi G, Cesta A, Ospedale di Avezzano

Bravi S, Biagioni F, Ospedale Citta’ di Castello; Acito L, Giustini L, Bascioni R, Ospedale di Fermo; Marzola M, Lelli G, Modonesi C Ospedale di Ferrara, Luppi G, Zironi S, Frassoldati A. Ospedale di Modena; Ardizzoia A, Barni S, Ospedale di Monza, Leonardi F, Pucci F, Camisa R, Ospedale di Parma; Tonato M, Corgna E, Ospedale di Perugia, Rodino’ C, Cavanna L, Ospedale di Piacenza; Gasperoni S, Ospedale di Terni; RosaBian A, Bassan F, Ospedale di Thiene

Attardo G, Cavalli C, Olgiati A, Ospedale di Vigevano, Conte P, Pfanner E, Brunetti I, Lencioni M, Ospedale S. Anna, Pisa; Campora E, Gatti C, Guarnieri D, Ospedale S. Remo

Gallo L, Caroti C, Mammoliti S, Ospedale Galliera Genova; Barsanti G, Battistoni M, Ospedale di Lucca; Brema F, Ospedale S, Paolo Savona, Taviani E, Ospedale di Empoli; Galli C, Ospedale di Livorno; Francesconi D, Ospedale di Viareggio; Spinelli I, Ospedale di Carrara; Chiavistelli P, Ospedale di Rosignano

